# A Testing Campaign Intervention Consisting of Peer-Facilitated Engagement, Point-of-Care HCV RNA Testing, and Linkage to Nursing Support to Enhance Hepatitis C Treatment Uptake among People Who Inject Drugs: The ETHOS Engage Study

**DOI:** 10.3390/v14071555

**Published:** 2022-07-16

**Authors:** Anna Conway, Heather Valerio, Maryam Alavi, David Silk, Carla Treloar, Behzad Hajarizadeh, Alison D. Marshall, Marianne Martinello, Andrew Milat, Adrian Dunlop, Carolyn Murray, Bianca Prain, Charles Henderson, Janaki Amin, Phillip Read, Pip Marks, Louisa Degenhardt, Jeremy Hayllar, David Reid, Carla Gorton, Thao Lam, Michael Christmass, Alexandra Wade, Mark Montebello, Gregory J. Dore, Jason Grebely

**Affiliations:** 1 The Kirby Institute, UNSW Sydney, Sydney, NSW 2052, Australia; hvalerio@kirby.unsw.edu.au (H.V.); msalehialavi@kirby.unsw.edu.au (M.A.); dsilk@kirby.unsw.edu.au (D.S.); bhajarizadeh@kirby.unsw.edu.au (B.H.); amarshall@kirby.unsw.edu.au (A.D.M.); mmartinello@kirby.unsw.edu.au (M.M.); pmarks@kirby.unsw.edu.au (P.M.); gdore@kirby.unsw.edu.au (G.J.D.); jgrebely@kirby.unsw.edu.au (J.G.); 2 Centre for Social Research in Health, UNSW Sydney, Sydney, NSW 2052, Australia; c.treloar@unsw.edu.au; 3 Centre for Epidemiology and Evidence, NSW Health, Sydney, NSW 2065, Australia; andrew.milat@health.nsw.gov.au; 4 Hepatitis NSW, Surry Hills, NSW 2010, Australia; adrian.dunlop@health.nsw.gov.au; 5 Drug and Alcohol Clinical Services, Hunter New England Local Health District, Newcastle, NSW 2300, Australia; 6 Population Health Strategy & Performance, NSW Health, Sydney, NSW 2065, Australia; carolyn.murray@health.nsw.gov.au (C.M.); bianca.prain@health.nsw.gov.au (B.P.); 7 NSW Users and AIDS Association, Surry Hills, NSW 2010, Australia; charlesh@nuaa.org.au; 8 Department of Health Systems and Populations, Macquarie University, Sydney, NSW 2109, Australia; janaki.amin@mq.edu.au; 9 Kirketon Road Centre, Sydney, NSW 2010, Australia; phillip.read1@health.nsw.gov.au; 10 National Drug and Alcohol Research Centre, UNSW Sydney, Sydney, NSW 2052, Australia; l.degenhardt@unsw.edu.au; 11 Alcohol and Drug Service, Metro North Mental Health, Metro North Hospital and Health Service, Brisbane, QLD 4029, Australia; jeremy.hayllar@health.qld.gov.au; 12 Drug and Alcohol Service, Illawarra Shoalhaven Local Health District, Wollongong, NSW 2500, Australia; david.reid1@health.nsw.gov.au; 13 Cairns Sexual Health Service, Cairns, QLD 4870, Australia; carla.gorton@health.qld.gov.au; 14 Drug Health, Western Sydney Local Health District, Sydney, NSW 2145, Australia; thao.lam@health.nsw.gov.au; 15 Next Step Community Alcohol and Drug Service, Perth, WA 6004, Australia; michael.christmass@mhc.wa.gov.au; 16 National Drug Research Institute, Curtin University, Perth, WA 6102, Australia; 17 Mid North Coast Liver Clinic, Mid North Coast Local Health District, Coffs Harbour, NSW 2450, Australia; alexandra.wade@health.nsw.gov.au; 18 Drug and Alcohol Services, Mid North Coast Local Health District, Coffs Harbour, NSW 2450, Australia; 19 North Sydney Local Health District, Sydney, NSW 2077, Australia; mark.montebello@health.nsw.gov.au

**Keywords:** direct-acting antiviral era, Hepatitis C virus elimination, Hepatitis C virus infection, Hepatitis C virus treatment, people who inject drugs

## Abstract

This study evaluated HCV treatment initiation among people who inject drugs (PWID) following an intervention of campaign days involving peer connection, point-of-care HCV RNA testing, and linkage to nursing support. ETHOS Engage is an observational cohort study of PWID attending 25 drug treatment clinics and needle and syringe programs in Australia (May 2018–September 2019). Point-of-care results were provided to the nurse, facilitating confirmatory testing and treatment. The study aimed to evaluate treatment uptake and factors associated with treatment at 24 months post-enrolment. There were 317 people with current HCV infection and eligible for treatment (median age 43, 65% male, 15% homeless, 69% receiving opioid agonist treatment, 70% injected in last month). Overall, 15% (47/317), 27% (85/317), 38% (120/317), and 49% (155/317) of people with current HCV infection had initiated treatment at 3-, 6-, 12-, and 24-months following testing, respectively. Homelessness (adjusted hazard ratio (aHR): 0.40; 95% confidence interval: 0.23, 0.71) and incarceration in the past 12 months (vs. never, aHR:0.46; 0.28, 0.76) were associated with decreased treatment initiation in the 24 months post-enrolment. This testing campaign intervention facilitated HCV treatment uptake among PWID. Further interventions are needed to achieve HCV elimination among people experiencing homelessness or incarceration.

## 1. Introduction

As part of the Global Health Sector Strategy on Viral Hepatitis 2016–21, the World Health Organisation aims to eliminate hepatitis C (HCV) as a major public health threat by 2030 [1]. Reaching the target of treating 80% of eligible people diagnosed with chronic HCV requires global and targeted efforts to improve access to testing and treatment services [1]. Globally, an estimated 6.1 million people who inject drugs were living with HCV in 2019 [2]. The criminalisation of people who use drugs increases the stigma around drug use and HCV [3], which may dissuade people from initiating HCV treatment [4]. People who inject drugs may receive suboptimal care due to multiple barriers including stigma, housing, criminalisation, and burdensome treatment pathways [5]. Novel interventions are needed to ensure PWID can access HCV testing and treatment [1]. Australia is uniquely placed to achieve HCV elimination given that highly curative direct-acting antiviral (DAA) therapy has been available since March 2016, regardless of virus acquisition and with no restrictions based on drug and alcohol use [6]. The advent of unrestricted access to DAAs is promising, yet recent work demonstrates that inequities in treatment uptake among PWID persist [7,8].

Complex HCV diagnostic pathways require multiple visits and can be difficult to navigate for people seeking HCV care [9]. Embedding HCV testing and treatment within services regularly used by PWID can increase access to testing and opportunities to initiate treatment [10]. Campaign days, where staff are deployed to screen large numbers of people as part of an event, have been used globally and show promise in improving testing and linkage to care [11,12]. Studies have evaluated HCV treatment uptake among PWID [7,8], but there is a need for research evaluating novel models of care in the DAA era [13]. Rapid diagnosis and treatment of HCV can reduce onward transmission and prevent progression of liver disease [14]. Understanding factors associated with delayed treatment uptake can highlight sub-populations of people who inject drugs who face greater barriers to care and can also facilitate the design of interventions to achieve HCV elimination.

The ETHOS Engage Study recruited a national cohort of people who inject drugs from opioid agonist treatment and needle and syringe programs during an era of ongoing provision of unrestricted HCV DAA treatment. At baseline, 24% of participants had current HCV infection and current infection was associated with homelessness, recent incarceration, and daily drug injection [7]. The study also found that 66% of people with previous chronic or current HCV had ever been treated [7].

This study extends the previously published observational study [7], to longitudinally evaluate the interventional component of ETHOS Engage which integrated peer connection, point-of-care HCV RNA testing, and nurse-led linkage to care delivered through screening and linkage to care campaign days. All the components were integrated on the campaign day to provide engagement with testing and linkage to care for HCV within a service providing opioid agonist treatment or NSP (as its primary function). The primary aim of this study was to evaluate treatment uptake among people diagnosed with current HCV infection following the intervention. The secondary aims were to evaluate factors associated with treatment uptake and report the estimated proportion of people with previous chronic or current HCV who initiated treatment in the 24 months following the intervention.

## 2. Materials and Methods

### 2.1. Study Design, Setting, and Participants

The ETHOS Engage Study is an observational cohort study [15]. An intervention is embedded within the study, aiming to enhance HCV screening, diagnosis, and linkage to care. Participants were recruited between May 2018–September 2019 from drug treatment clinics (*n* = 21) and needle and syringe programs (NSPs) (*n* = 4) in four Australian States: New South Wales (*n* = 17), Queensland (*n* = 4), South Australia (*n* = 2), and Western Australia (*n* = 2).

Inclusion criteria were informed consent, ≥18 years of age, history of injecting drug use, and either injecting drug use in the previous six months or current opioid agonist treatment (OAT). Pregnant women were excluded given that FibroScan^®^ (Echosens, Paris, France) was contraindicated at time of study protocol approval. The study protocol was approved by the Human Research Ethics Committees at St Vincent’s Hospital, Sydney and the Aboriginal Health and Medical Research Council (HREC Ref: HREC/17/SVH/113).

### 2.2. Procedures

ETHOS Engage campaign days were advertised using posters (Supplementary Figure S1), cards distributed with injecting equipment, and by word of mouth. Recruitment spanned one to five days at each site and included a team of university staff, peer workers specialised in either HCV or injecting drug use, and clinic personnel. The campaign days were embedded within primary operation of drug treatment clinics and NSPs, without appointments, allowing for opportunistic engagement of participants attending the site for standard services.

The interventional component of ETHOS Engage consisted of multiple stages. People attending the clinic were approached by peer workers who informed them about the study, providing transparent information about the implications of study participation. On each campaign day, one peer was working on-site to initiate the recruitment of study participants and to provide education about point-of-care HCV testing and treatment. The peer worker offered the opportunity for people to discuss any HCV-related concerns before agreeing to participate in the study. If the person was eligible to participate and gave informed consent, a 100 µL finger-stick capillary whole-blood was collected to test for HCV RNA using the point-of-care Xpert HCV Viral Load Fingerstick Assay (Cepheid, Sunnyvale, CA, USA; lower limit of quantification 100 IU/mL, upper limit of quantification 10^8^ log10 IU/mL; 100% sensitivity, 100% specificity) [9]. Baseline data were collected by participants using a self-administered computer tablet-based questionnaire. This data included demographics, behavioural risk, and HCV history (testing, infection status, and treatment). Liver fibrosis stage was assessed using transient elastography (FibroScan^®^, Echosens, Paris, France) with a lower and upper detection limit of 2.5 and 75 kPa, respectively. Participants then underwent a brief consultation with clinical staff. Participation was compensated with a shopping voucher (AUD$30) for their time and effort in participating. 

At the time of the study, the Australian Therapeutics Goods Administration had not yet approved the Xpert HCV Viral Load Fingerstick assay and so HCV RNA test results could not be provided to participants in the same visit. Results were returned to clinics or programs after in-house quality assurance checks. Clinic or service staff were asked to facilitate confirmatory HCV testing via venous blood draw (not necessarily on the same day as point-of-care testing), communicate results from confirmatory HCV testing, and facilitate treatment initiation. Clinics employed nurse-led linkage to care but this was not standardised and so strategies employed by the clinics were heterogenous.

Follow-up data were collected at the site level. Participants were not asked to attend for follow-up visits as part of the study and data were collected during routine clinic visits. A standardised online case report form was completed by clinic nurses based on a medical record review at 12- and 24-months post-enrolment. This was completed for each participant who had HCV infection at the time of enrolment, including data on HCV treatment initiation and loss to follow-up.

### 2.3. Outcomes

The primary outcome was HCV treatment initiation following diagnosis of current HCV infection in ETHOS Engage (detected with an HCV RNA assay). Treatment initiation was assessed at 3-, 6-, 12-, and 24-months following detection of current HCV infection. The secondary outcome was time to initiation of HCV treatment within the 24-month period following diagnosis with HCV infection in ETHOS Engage. 

### 2.4. Statistical Analysis

Demographic and behavioural factors hypothesised to be associated with HCV treatment initiation were determined using previously published results from ETHOS Engage [7,8] and included: (i) age at survey; (ii) gender (male, female, other); (iii) Aboriginal or Torres Strait Islander; (iv) homelessness (when asked where they had spent the majority of nights in the past six months, participants responded no usual residence/shelter/squat); (v) currently receiving OAT (no/yes); (vi) incarceration history (never/more than 12 months ago/in last 12 months); (vii) injection drug use within the last month (no/yes); (viii) hazardous alcohol consumption (defined by AUDIT-C [16]); (ix) liver fibrosis stage (liver stiffness measurement <7.0 kpa no significant fibrosis [F0/F1] or ≥7.0 kpa significant fibrosis [≥F2]) [17]. Participants with no FibroScan score or invalid results were classified as “Unknown”, and (x) if ever diagnosed with HCV self-report at enrolment (no/yes, never treated/yes, ever treated).

People who had initiated HCV treatment in the 12 weeks prior to enrolment, with no further treatment initiation recorded post-enrolment, were not considered “at-risk” and were excluded from treatment uptake analyses. Observation time for treatment initiation commenced on date of ETHOS Engage enrolment (i.e., date of HCV RNA test) and ended on date of HCV treatment prescription, date of death, date reported by clinic site as lost to follow-up, or 24 months post enrolment in ETHOS Engage, whichever occurred first. Participants who were reported as lost to follow-up who had missing information on date of loss to follow-up, were censored at the time of the reporting. The cumulative proportion of participants who initiated treatment at each timepoint was reported along with 95% confidence intervals, using the total sample as the denominator. Kaplan Meier estimates were used to report cumulative probability of HCV treatment initiation over time, with 95% confidence intervals. Cox regression models were used to identify factors associated with time to HCV treatment initiation, giving crude and adjusted hazard ratios (crude HR and adjusted HR). Variables with *p* value < 0.1 in the univariate Cox regression models were retained in the multivariate model.

Evidence was based on the self-reported history of HCV treatment among participants with either previous (self-reported history of HCV treatment) or current HCV infection (in participants who have been treatment eligible) [8]). All analyses were conducted using Stata 14.0 (StataCorp, College Station, TX, USA).

## 3. Results

### 3.1. Sample Characteristics

Overall, 1443 participants were recruited between May 2018 and September 2019, of whom 1388 had a HCV RNA point-of-care test result (Supplementary Figure S2). Among people with a HCV RNA test result, 24% (*n* = 331) had current HCV infection. People reporting HCV treatment in the 12 weeks prior to enrolment who were RNA positive and had no post-enrolment treatment initiation were excluded (4%, *n* = 14), leaving 317 people with current HCV infection eligible for treatment (Supplementary Table S1). Compared to people without current HCV infection, people with current HCV infection had a higher proportion of homelessness (15% vs. 9%, *p* = 0.002), incarceration > 12 months before enrolment (61% vs. 48%, *p* < 0.001), to have injected drugs in the last month (70% vs. 61%, *p* = 0.006), and to have advanced liver disease (FibroScan; liver stiffness measurement > 7.0 kpa; 36% vs. 19%, *p* < 0.001) (Supplementary Table S1).

### 3.2. Prevalence and Characteristics of HCV Treatment Initiation at Three Months Post-Enrolment

Of 317 people with current HCV infection eligible for treatment, 15% (*n* = 47, 95% confidence interval (CI): 11–19%) had initiated treatment at three months. At three months post-enrolment, treatment initiation was lower among people who were homeless (4% vs. 17%, *p* = 0.002), people incarcerated in last 12 months (vs. more than 12 months ago or no incarceration history; 8% vs. 16% and 19%, *p* = 0.165), people who injected drugs in the last month (14% vs. 16%, *p* = 0.792), and people who had never previously been diagnosed with HCV (8% vs. 15% amongst those diagnosed but never treated and 21% among those previously treated, *p* = 0.150) (Table 1). Overall, 15% (47/317, 95% CI: 11–19%), 27% (85/317, 95% CI 22–32%), 38% (120/317, 95% 32–43%), and 49% (155/317, 95% CI 43–55%) of people with current HCV infection had initiated treatment at 3-, 6-, 12-, and 24-months following testing, respectively.

### 3.3. Factors Associated with Time to HCV Treatment Initiation

For people diagnosed with current HCV infection, the median follow-up was 365 days (IQR: 171–395 days). For people who initiated HCV treatment at 24 months post-enrolment (*n* = 155), the median time between testing and treatment initiation was 169 days (IQR: 73–335 days) or around six months.

**Figure 1:**
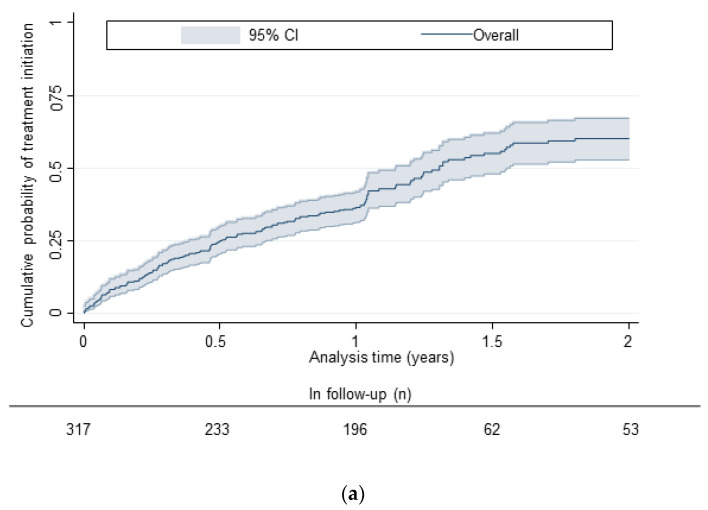

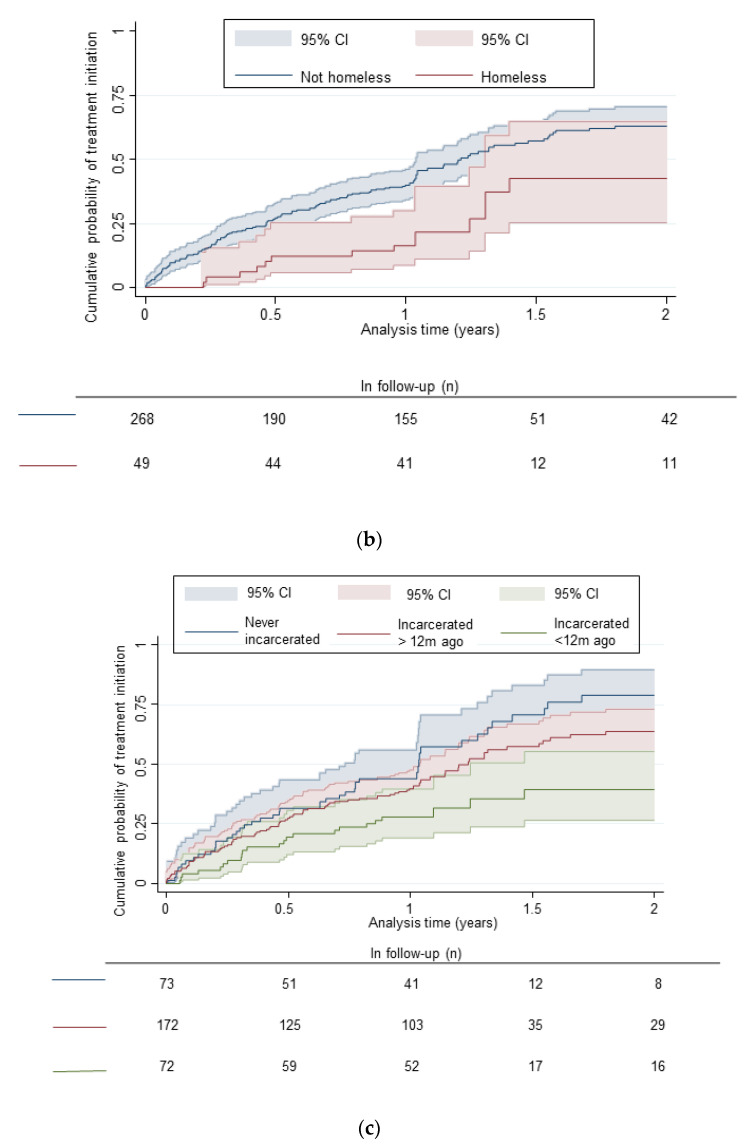
(**a**) Kaplan-Meier curves depicting estimated time (years) to DAA treatment initiation among people diagnosed with current HCV infection in ETHOS Engage overall. (**b**) Kaplan-Meier curves depicting estimated time (years) to DAA treatment initiation among people diagnosed with current HCV infection in ETHOS Engage by homelessness. (**c**) Kaplan-Meier curves depicting estimated time (years) to DAA treatment initiation among people diagnosed with current HCV infection in ETHOS Engage by history of incarceration.

In the Kaplan Meier estimates, among all those diagnosed with current HCV infection, the cumulative probabilities of HCV treatment initiation were 15% (95% CI: 12%–20%), 27% (95% CI: 22%–32%), 38% (95% CI: 33%–44%), and 62% (95% CI: 55%–69%) at 3-, 6-, 12-, and 24-months following testing, respectively (Figure 1, Supplementary Table S2). Cumulative probability of treatment initiation by 24 months post-enrolment was lower in people with a history of incarceration (vs. no history of incarceration) and people who were homeless (vs. not) (Figure 1, Supplementary Table S2).

After adjusting for age and Aboriginal and Torres Strait Islander identification, homelessness (adjusted HR 0.40, 95% CI 0.23–0.71) and incarceration in the 12 months prior to enrolment (vs. no history of incarceration, adjusted HR 0.46, 95% CI 0.28–0.76) were associated with longer time to HCV treatment initiation (Table 2).

### 3.4. Progress towards Elimination Targets

Among everyone with a valid HCV RNA result at enrolment, 57% (788/1388) had evidence of previous or current HCV infection. Of those, 66% (520/788) reported having ever initiated HCV treatment. Adding people who initiated treatment in the 24 months post-enrolment, a total of 83% (652/788) of those ever eligible initiated HCV treatment.

## 4. Discussion

An intervention consisting of screening campaign days with peer connection, point-of-care HCV RNA testing, and nurse-led linkage to care, resulted in 15% of people with current HCV infection at enrolment initiating treatment within three months (27% at six months and 49% at 24 months). When combined with pre-intervention treatment, a total of 83% of participants with previous or current chronic HCV infection initiated HCV treatment, advancing progress to WHO HCV elimination targets [1]. The analysis of treatment uptake in the 24 months post-enrolment reveals disparities with lower treatment uptake observed among people who are homeless or recently incarcerated. These findings are consistent with previous analyses of factors associated with treatment uptake from ETHOS Engage [7,8], highlighting the need for tailored support to be made available in services which are frequently used by these populations.

Fingerstick point-of-care HCV RNA testing identified 317 people with current HCV infection who were eligible for treatment, with a cumulative probability of 27% initiating HCV treatment within six months of enrolment. This is comparable with data from New South Wales, Australia, where treatment uptake among people with drug dependence was estimated at 27% in the six months following diagnosis [18]. This suggests the HCV testing campaigns in ETHOS Engage facilitated an increased reach in HCV testing, by providing testing for people who would not have received it had this study not been performed. As such, it is not surprising that the proportion with treatment uptake was similar to standard of care given most people had to come back for multiple visits to either have confirmatory testing or treatment. The higher treatment uptake in the period immediately following diagnosis indicates the utility of running testing campaigns at frequent intervals, either annually or biannually. Using the fingerstick HCV RNA assay in the ETHOS Engage study allowed testing to take place on-site without venepuncture. The lack of Australian Therapeutics Goods Administration approval at the time of the study prevented results from being communicated on the same day, and the requirements to assess genotypes before initiating treatment obliged people to later have venepuncture for confirmatory testing. Studies providing HCV RNA point-of-care testing and same-day results to people who inject drugs have reported higher proportions of people initiating treatment in a needle and syringe program (74%) [19], medically supervised injecting sites (89%) [20], mobile outreach models (74%) [21], and prison (93%) [22]. This demonstrates the importance of offering single-visit testing and treatment to considerably increase HCV treatment uptake among populations of people who inject drugs. In the second recruitment wave of ETHOS Engage (2019–2021), the Xpert HCV Viral Load Fingerstick assay had been approved by the Therapeutics Goods Administration, so the results of point-of-care HCV RNA testing could be provided directly to participants and should lead to increased treatment uptake. The need for confirmatory testing via venepuncture will no longer be required, which may increase treatment uptake given a preference for finger-stick testing among people who inject drugs [23]. Follow-ups to assess treatment initiation after enrolment in the second recruitment wave of ETHOS Engage is ongoing.

Homelessness was associated with reduced HCV treatment uptake, consistent with previous studies [7,8]. Among people who were homeless, the cumulative probability of HCV treatment uptake was 42% at 24 months, compared to 62% overall. There are multiple mechanisms by which homelessness might impact treatment uptake including providers considering homelessness a sign of “unmanageability” [5], and people considering treatment initiation as less urgent than other, competing priorities [24,25]. Although opioid agonist treatment services and NSPs do not require people to be housed to access services, people experiencing homelessness may still face increased structural barriers to access [26]. Stable housing reduces the risk of HCV transmission [27] and removes stressors to allow people to prioritise health and wellbeing [28]. Some interventions have attempted to mitigate the effects of homelessness with regards to treatment uptake, by providing flexible appointments [26] or mobile testing and treatment [29]. A mobile unit employing a same day ‘test and treat’ model for a cohort in which the majority were experiencing homelessness, reported 77% initiating treatment [30]. Although there is weak evidence for the effectiveness of financial incentives to broadly increase HCV treatment uptake [13], high willingness to partake in such studies [31] indicates the need for further investigation. Less investigated are the additional system level barriers to providing incentives for treatment, such as provider or clinic reluctance.

History of incarceration was associated with reduced HCV treatment initiation. Other Australian studies found, in adjusted analyses, recent incarceration was not associated with treatment uptake [7,8,18] or was associated with increased treatment uptake [32]. Availability of HCV testing and treatment in Australian prisons and implementation of initiatives for treatment scale up in prison [33] may be improving treatment initiation among incarcerated people. In the current study, lower treatment uptake amongst people who have been incarcerated may be due to missing information on follow-up in this population, possibly due to reincarceration. Nevertheless, subgroups of people with recent history of incarceration may have poorer treatment uptake, such as people serving sentences that are shorter than the duration of treatment. Housing, mental health, social support, and economic hardship have been identified as structural factors which impact health service access upon release from prison [34] and likely influence HCV treatment initiation. Despite access to HCV care in Australian prisons, criminalisation and imprisonment is damaging to health and wellbeing. Criminalisation exacerbates the stigma experienced by people who inject drugs [3], which diminishes access to healthcare such as HCV treatment [4]. There is a need for more research to understand the period post-release to ensure people are supported to initiate or complete HCV treatment. There is evidence that patient navigation and transitional care coordination can mitigate structural barriers to initiating treatment for people being released from prison [35]. Decarceration as a public health strategy has had increased attention during the COVID-19 pandemic [36] and could improve outcomes for other infectious diseases including HCV.

This study has limitations. At the time of study, the test was not approved for diagnosis and so results were not provided to participants on the day of test, therefore not realising the full potential of HCV RNA point-of-care testing. Nevertheless, the incorporation of peers and the relatively quick return of results to the clinic represented an improvement upon current standard of care. Several of the variables in the Cox regression model are time varying by nature (e.g., homelessness, OAT, recent injecting drug use) but, because of the design of the study as an observational cohort, they are included in the model as fixed variables. In Wave 2, ETHOS Engage recruited a similar population which suggests minimal change in these variables over time [8]. There was no control group in this study, making it difficult to assess what outcomes would have been in the absence of this intervention. The study follow-up only collected data on treatment initiation, not cure or reinfection, which would likely have an impact on the achievement of the WHO target to treat 80% of persons diagnosed with chronic HCV infection. Recruitment mainly took place within opioid agonist treatment settings, potentially biasing our cohort to people who were already engaged with services and under-representing the population of PWID other than opioids. Clinics had autonomy in how to manage patient follow-up and care which may have impacted results. Services which require registration of patients (i.e., to provide OAT) may have had improved treatment uptake compared to low threshold service providers such as NSPs. Given the importance of embedding HCV care in services which are frequently used by people who inject drugs, same day ‘test and treat’ models are likely to be particularly important to reduce loss to follow-up in low threshold services like NSPs. The analysis made no distinction between people lost to follow-up and people who had moved services or been imprisoned, who may remain engaged with care and have higher treatment uptake. Finally, the ETHOS Engage survey did not account for mental health comorbidities and inpatient hospitalisation, factors that have been associated with lower treatment uptake [33,37].

These results have implications for public health. This screening and linkage to care campaign day intervention brings low threshold testing and treatment to services utilised by PWID, improving accessibility and reducing opportunities for stigma [38]. Peer workers are integral to the ETHOS model, connecting with people at risk of HCV with information to make informed decisions on healthcare [39]. Peer workers allow participants to discuss HCV testing and treatment without impacting the therapeutic relationship with OAT providers [40]. This study supports findings that integrated care can be effective at increasing treatment uptake [13]. In addition, venepuncture is a barrier to testing for people who inject drugs, recognised amongst both practitioners [41] and patients [25]. The decentralisation of diagnostics and utilisation of innovative diagnostic technology (including point-of-care and dried blood spot testing) is acceptable among people who inject drugs [23], has been shown to increase testing [13], and will be key in reducing drop-off along the HCV care cascade. Event-based models of testing and treatment, or the use of campaign days, have been successful in engaging large numbers of people at risk of HCV [42]. Further research is needed to evaluate effectiveness and cost-effectiveness of these interventions, particularly with availability of novel diagnostic approaches that facilitate single-visit test and treat strategies. Traditional models of care and wider structural barriers may prevent people who are homeless or have a history of incarceration from initiating treatment compared with others. Criminalisation of drug possession [43] and lack of appropriate housing [44] are detrimental to health and access to health services, likely slowing progress to HCV elimination.

## 5. Conclusions

HCV screening and linkage to care campaign days, including peer connection, point-of-care HCV RNA testing, and nurse-led linkage to care in drug treatment clinics, were facilitated by HCV treatment initiation in people who inject drugs. Although the proportion of treatment initiation was similar to population-based estimates, the campaign day model may have improved the reach of testing for people who would otherwise not have tested and treated for HCV. Building upon the 66% of eligible ETHOS Engage participants who had ever received HCV treatment at enrolment, 24 months post-intervention, that proportion rose to 83%. Importantly, this result surpasses WHO elimination goals in this population. Offering same-day results to facilitate linkage to care could be an important strategy to improve the impact of a similar intervention. Further studies are needed to evaluate the effectiveness and cost-effectiveness of HCV screening and linkage to care campaign days. Homelessness and incarceration history are associated with lower treatment uptake—highlighting the crucial message that people who inject drugs require tailored support in accessing and succeeding in HCV treatment. Public health responses that address housing, decarceration, and decriminalisation of drugs will be critical in reducing barriers to care and advancing progress towards HCV elimination.

## Figures and Tables

**Table 1:** Baseline characteristics of people with HCV infection, by treatment initiation status three months following HCV diagnosis in ETHOS Engage (*n* = 317).

Characteristic		Current HCV Infection	No Treatment Initiation within Three Months or Lost to Follow-Up	Initiated Treatment Three Months Post Diagnosis	
		*n* (col%)	*n* (% of Current HCV Infection)	*n* (% of Current HCV Infection)	*p* Value
Total (N)		317 (100%)	270 (85%)	47 (15%)	
Age at enrolment	<45	184 (58%)	159 (86%)	25 (14%)	
	≥45	133 (42%)	111 (83%)	22 (17%)	0.465
Gender	Male	205 (65%)	177 (86%)	28 (14%)	
	Female	110 (35%)	91 (83%)	19 (17%)	
	Other	2 (1%)	2 (100%)	0 (0%)	0.580
Aboriginal or Torres Strait Islander	No	241 (76%)	204 (85%)	37 (15%)	
	Yes	76 (24%)	66 (87%)	10 (13%)	0.639
Homeless	No	268 (85%)	223 (83%)	45 (17%)	
	Yes	49 (15%)	47 (96%)	2 (4%)	0.021
Currently receiving OAT	No	98 (31%)	80 (82%)	18 (18%)	
	Yes	219 (69%)	190 (87%)	29 (13%)	0.235
Incarceration history	Never	73 (23%)	59 (81%)	14 (19%)	
	More than 12 months ago	172 (54%)	145 (84%)	27 (16%)	
	In last 12 months	72 (23%)	66 (92%)	6 (8%)	0.165
Recency of injecting	More than a month ago	96 (30%)	81 (84%)	15 (16%)	
	Within last month	221 (70%)	189 (86%)	32 (14%)	0.792
Hazardous alcohol consumption ^†^	No	188 (59%)	160 (85%)	28 (15%)	
	Yes	127 (40%)	108 (85%)	19 (15%)	0.839
Fibrosis -Fibroscan result (kpa)	<7.0	184 (58%)	154 (84%)	30 (16%)	
	>7.0	115 (36%)	101 (88%)	14 (12%)	
	Unknown	18 (6%)	15 (83%)	3 (17%)	0.604
	No	61 (19%)	56 (92%)	5 (8%)	
Diagnosed with HCV prior to study	Yes, never treated	204 (64%)	173 (85%)	31 (15%)	
	Yes, ever treated	52 (16%)	41 (79%)	11 (21%)	0.150

† Excluding people who did not identify as men or women (*n* = 2). Acronyms–OAT: opioid agonist treatment, HCV: hepatitis C virus. *p* value based on chi-square test of differences.

**Table 2:** Cox regression–factors associated with treatment initiation at 24 months post-diagnosis (*n* = 317).

Characteristic		Person-Years Observation	Incidence Rate	Unadjusted Hazard Ratio (95% CI)	Adjusted Hazard Ratio (95%CI)
Age at enrolment	Year			1.30 (0.95–1.78)	1.00 (0.98–1.01)
Gender	Male	199	0.48	Ref	
	Female	104	0.54	1.12 (0.80–1.55)	
	Transgender	1	2.00	2.88 (0.71–11.73)	
Aboriginal or Torres Strait Islander	No	226	0.55	Ref	Ref
	Yes	79	0.37	0.67 (0.45–1.00)	0.66 (0.44–0.99)
Homeless	No	247	0.57	Ref	Ref
	Yes	57	0.23	0.41 (0.23–0.72)	0.40 (0.23–0.71)
Currently receiving OAT	No	94	0.41	Ref	
	Yes	211	0.54	1.29 (0.90–1.86)	
Incarceration history	Never	64	0.70	Ref	Ref
	More than 12 months ago	164	0.52	0.77 (0.54–1.10)	0.83 (0.58–1.19)
	In last 12 months	77	0.30	0.44 (0.27–0.73)	0.46 (0.28–0.76)
Recency of injecting	More than a month ago	98	0.58	Ref	
	Within last month	206	0.47	0.84 (0.61–1.17)	
Hazardous alcohol consumption ^†^	No	183	0.49	Ref	
	Yes	120	0.52	1.07 (0.78–1.48)	
Fibrosis-Fibroscan result (kpa)	<7.0	171	0.54	Ref	
	>7.0	116	0.46	0.84 (0.60–1.17)	
	Unknown	18	0.44	0.82 (0.40–1.69)	

† Excluding people who did not identify as men or women (*n* = 2). Acronyms–OAT: opioid agonist treatment, HCV: hepatitis C virus, CI: confidence interval.

## Data Availability

Not applicable.

## Supplementary Materials

The following supporting information can be downloaded at: https://www.mdpi.com/article/10.3390/v14071555/s1, Figure S1: Promotional material to advertise ETHOS Engage study prior to recruitment; Figure S2: ETHOS Engage participant flowchart, current HCV status and treatment uptake at 24 months post-enrolment (*n* = 1388); Table S1: Characteristics of study population according to current HCV infection (*n* = 1374); Table S2: Cumulative probability of treatment initiation by 12 months and 24 months following HCV diagnosis.
