# Supplementary material for: A Testing Campaign Intervention Consisting of Peer-Facilitated Engagement, Point-of-Care HCV RNA Testing, and Linkage to Nursing Support to Enhance Hepatitis C Treatment Uptake among People Who Inject Drugs: The ETHOS Engage Study
Source: Viruses. 2022 Jul 16;14(7):1555. doi: 10.3390/v14071555 (PMC9316739; doi:10.3390/v14071555)
Supplement: Supplementary file 1 [file viruses-14-01555-s001.zip › viruses-1783087-supplementary.pdf]

Supplementary Figure S1. Promotional material to advertise ETHOS Engage study prior to recruitment.

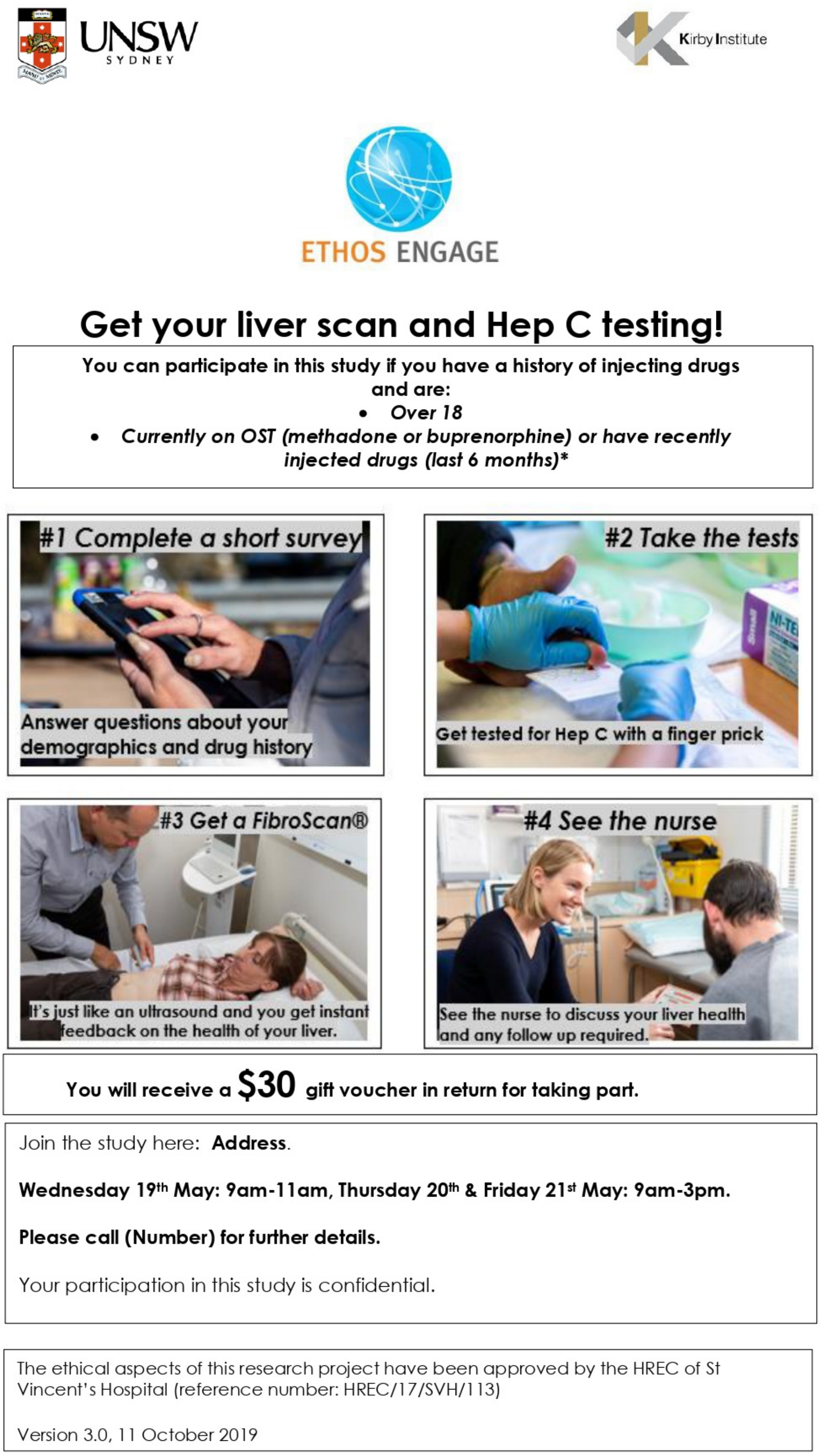

Supplementary Figure S2. ETHOS Engage participant flowchart, current HCV status and treatment uptake at 24 months post-enrolment (N = 1388).

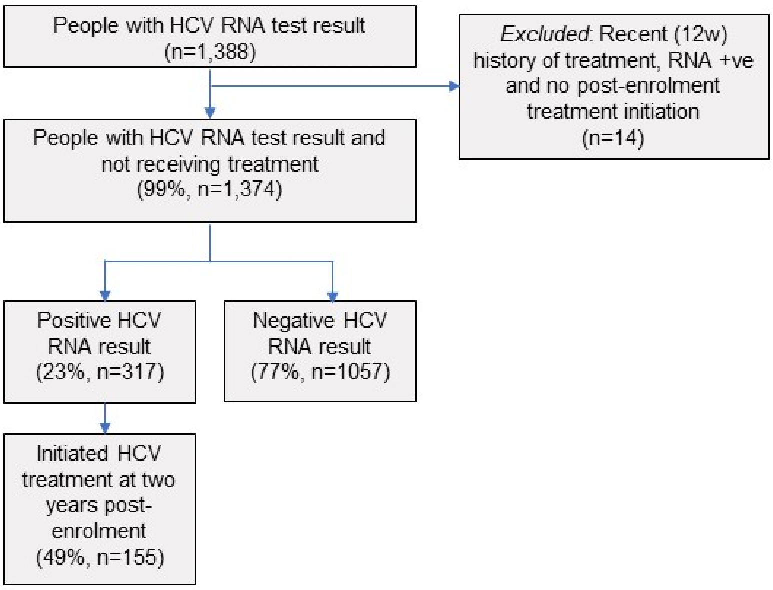

Supplementary Table S1. Characteristics of study population according to current HCV infection (N=1374).

Characteristic		All	No current HCV infection	Current HCV infection
		N (col%)	n (col%)	n (col%)
Total (N)		1374 (100%)	1057 (100%)	317 (100%)
Age at enrolment	Median (IQR)	43 (37-50)	43 (37-50)	43 (37-49)
Gender	Male	880 (64%)	675 (64%)	205 (65%)
	Female	491 (36%)	381 (36%)	110 (35%)
	Other	3 (0%)	1 (0%)	2 (1%)
Aboriginal or Torres Strait Islander	No	1052 (77%)	811 (77%)	241 (76%)
	Yes	322 (23%)	246 (23%)	76 (24%)
Homeless	No	1227 (89%)	959 (91%)	268 (85%)
	Yes	147 (11%)	98 (9%)	49 (15%)
Currently receiving OAT	No	387 (28%)	289 (27%)	98 (31%)
	Yes	987 (72%)	768 (73%)	219 (69%)
Incarceration history	Never	451 (33%)	378 (36%)	73 (23%)
	More than 12 months ago	678 (53%)	506 (48%)	172 (61%)
	In last 12 months	245 (14%)	173 (16%)	72 (16%)
Recency of injecting	More than a month ago	506 (37%)	410 (39%)	96 (30%)
	Within last month	868 (63%)	647 (61%)	221 (70%)
Hazardous alcohol consumption†	No	871 (63%)	683 (65%)	188 (59%)
	Yes	500 (36%)	373 (35%)	127 (40%)
Fibrosis - Fibroscan result (kpa)	<7.0	966 (70%)	782 (74%)	184 (58%)
	>7.0	320 (23%)	205 (19%)	115 (36%)
	Unknown	88 (6%)	70 (7%)	18 (6%)

† Excluding people who did not identify as men or women (n=2)
Acronyms – OAT: opioid agonist treatment, HCV: hepatitis C virus

Supplementary Table S2. Cumulative probability of treatment initiation by 12 months and 24 months following HCV diagnosis.

		Follow up time, years (95% CI)	
		1	2
Overall		38% (33%-44%)	62% (55%-69%)
Age at enrolment	<45	35% (29%-43%)	58% (49%-68%)
	≥45	42% (34%-51%)	67% (57%-77%)
Gender	Male	38% (32%-45%)	58% (50%-67%)
	Female	37% (29%-47%)	69% (57%-80%)
	Other	-	-
Aboriginal or Torres Strait Islander	No	41% (35%-47%)	66% (58%-74%)
	Yes	30% (21%-42%)	50% (36%-66%)
Homeless	No	42% (37%-48%)	66% (58%-73%)
	Yes	16% (9%-30%)	42% (25%-65%)
Currently receiving OAT	No	31% (23%-41%)	58% (44%-73%)
	Yes	42% (35%-48%)	64% (56%-72%)
Incarceration history	Never	45% (35%-57%)	78% (65%-89%)
	More than 12 months ago	40% (33%-47%)	64% (54%-73%)
	In last 12 months	28% (19%-40%)	39% (26%-55%)
Recency of injecting	More than a month ago	41% (32%-51%)	67% (57%-78%)
	Within last month	37% (31%-44%)	59% (49%-68%)
Hazardous alcohol consumption[†]	No	38% (32%-46%)	59% (50%-68%)
	Yes	37% (29%-46%)	67% (55%-78%)
Fibrosis -Fibroscan result (kpa)	<7.0	45% (38%-54%)	63% (54%-73%)
	>7.0	31% (24%-40%)	61% (50%-72%)
	Unknown	28% (13%-54%)	64% (36%-90%)

[†] Excluding people who did not identify as men or women (n=2)
Acronyms – OAT: opioid agonist treatment, HCV: hepatitis C virus, CI: confidence interval
